# A typically progressive dissection of the internal carotid artery with recurrent hiccups: A case report with continuous 2‐year data recording

**DOI:** 10.1002/ibra.12074

**Published:** 2022-11-09

**Authors:** Zong‐Min Zhang, Xin‐Xin Yang, Li Ling, Man‐Hong Zhou

**Affiliations:** ^1^ Department of Geriatrics Affiliated Hospital of Zunyi Medical University Zunyi Guizhou China; ^2^ Department of Anesthesiology, Union Hospital, Tongji Medical College Huazhong University of Science and Technology Wuhan China; ^3^ Department of Emergency KweiChow Moutai Hospital Renhuai Guizhou China; ^4^ Department of emergency Affiliated Hospital of Zunyi Medical University Zunyi Guizhou China

**Keywords:** digital subtraction angiography (DSA), internal carotid artery dissection (ICAD), magnetic resonance imaging (MRI), recurrent hiccups

## Abstract

Patients with internal carotid artery dissection (ICAD) usually report headache, neck pain, Horner's syndrome, and ischemic stroke. Because the posterior cranial nerve is involved, some patients may show different forms of posterior cranial nerve paralysis. There have been no reports of patients with ICAD showing repeated hiccups. Here, to help clinicians identify ICAD early and gain a better understanding of the atypical manifestations of the disease, we report an atypical case of recurrent hiccup symptoms caused by ICAD.

## Graphical abstract

The dissection of the internal carotid artery resulted in an intermural hematoma, which resulted in stenosis of the vascular lumen and the shedding of the thrombi, resulting in symptoms of ischemic stroke; this is a common clinical manifestation. Some patients may present with different forms of posterior cranial nerve palsy due to the involvement of the posterior cranial nerves. There have been no reports of symptoms of repeated hiccups. The vagus nerve is in close contact with the internal carotid artery in the carotid sheath, when ICAD occurs, it may affect the adjacent vagus nerve. Sandwich intramural hematoma may lead to the expansion of the blood vessel lumen to stimulate the vagus nerve, and especially when the neck is rotated, the vagus nerve is further pulled and stimulated, resulting in repeated hiccups. Hiccups are probably an atypical presentation of internal carotid artery dissection that should be a concern for clinicians.

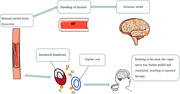

## 1 INTRODUCTION

Internal carotid artery dissection (ICAD) refers to an intramural hematoma or aneurysm‐like dilatation caused by internal carotid artery intimal tear and blood entering the blood vessel wall through intimal rupture, which leads to vascular stenosis, occlusion, or cerebral palsy.^1^, ^2^ ICAD is a common cause of stroke in young people,^3^, ^4^ which is rare in clinical practice. At the same time, some patients lack typical clinical manifestations in the early stage (ICAD triad: unilateral frontotemporal headache, neck pain, Horner's syndrome);^5^ therefore, it is difficult to identify and treat the disease in the early stage.^6^, ^7^ A case of atypical ICAD is reported in this paper. The patient, a 56‐year‐old female, was hospitalized in Affiliated Hospital of Zunyi Medical University from December 23, 2019 to January 20, 2020 due to acute cerebral infarction. After repeated hiccups during anticoagulant therapy, she was further diagnosed with ICAD. While receiving conservative treatment, the patient developed symptoms of transient ischemic attack (TIA) and underwent left internal carotid artery stenting. In this paper, the related literature is reviewed and the whole process of diagnosis of atypical ICAD is analyzed to provide useful suggestions for the early diagnosis of the disease.

## 2 CASE INFORMATION

**Figure 1:**
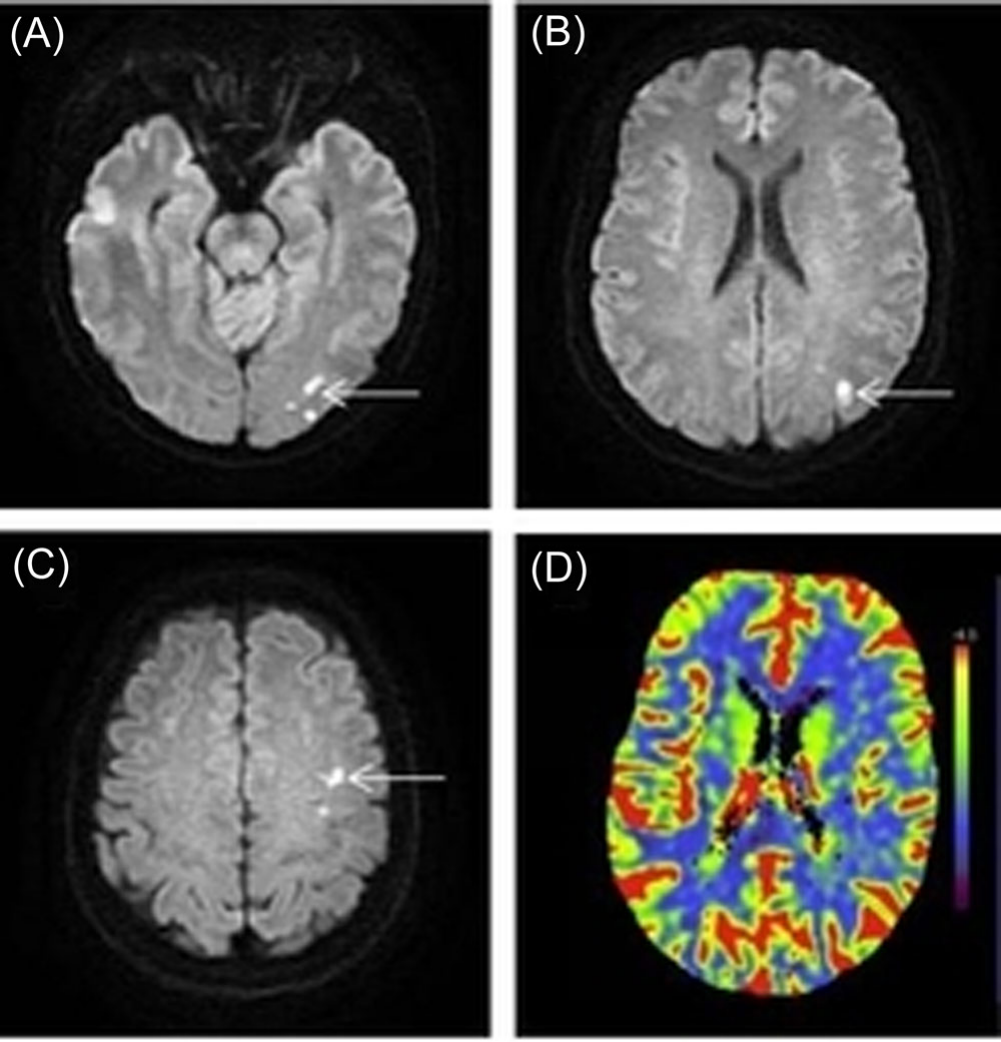
(A–C) Brain diffusion‐weighted imaging showing multiple acute cerebral infarction in the left occipital lobe (white arrow) and parietal lobe. (D) Cerebral perfusion imaging showing decreased perfusion in the left cerebral hemisphere. [Color figure can be viewed at wileyonlinelibrary.com]

**Figure 2:**
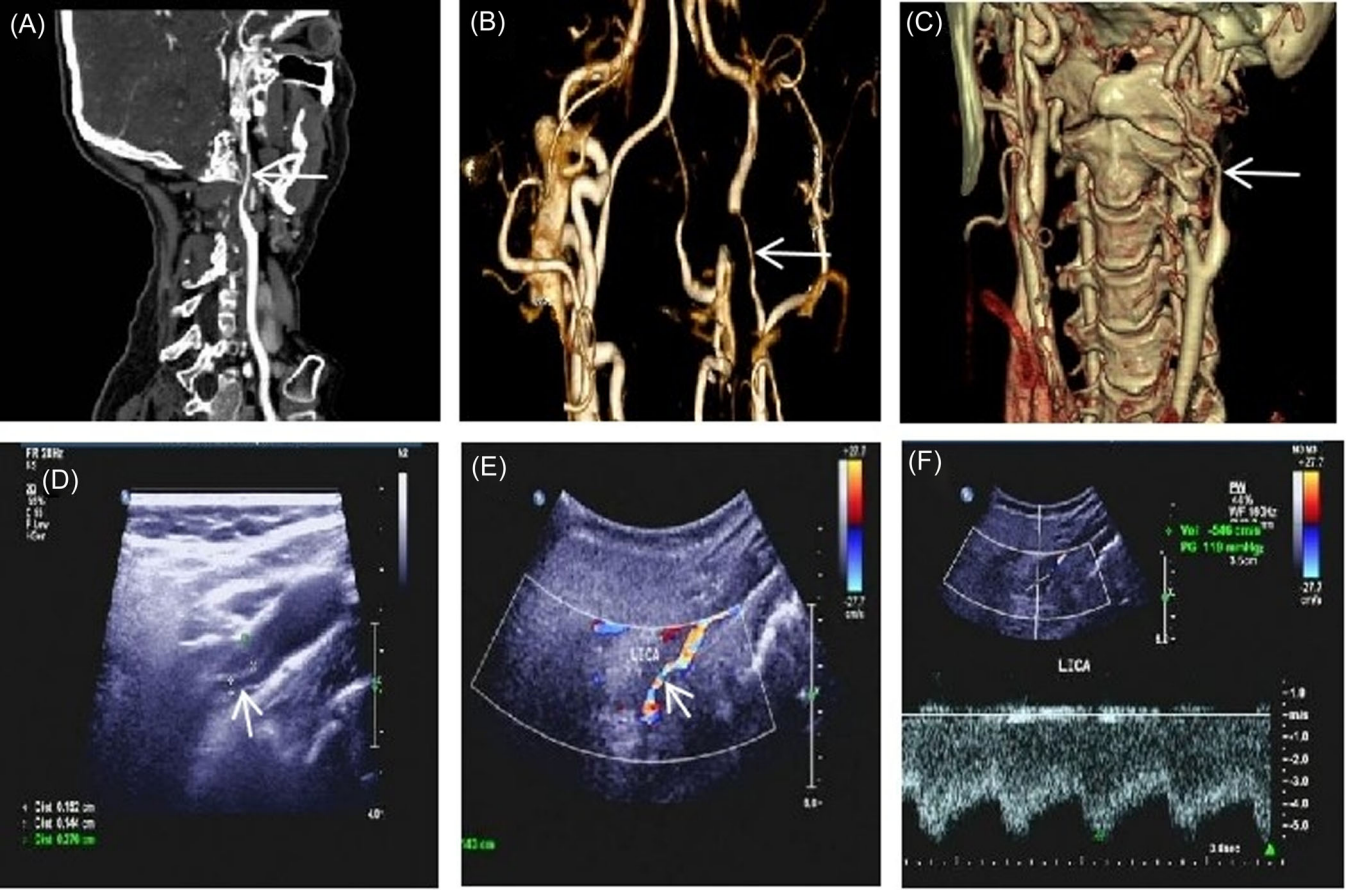
(A–C) Carotid computed tomography showing thin left internal carotid artery segment and critical stenosis of the lumen (white arrow). (D) Carotid ultrasound showing true and false double lumens in the left internal carotid artery, hypoechoic filling in the false lumen, and narrowing of the true lumen (white arrow); the residual inner diameter is about 1.4 mm and the original inner diameter was about 6.8 mm. (E) Color Doppler flow imaging examination showing no obvious blood flow signal in the false lumen, and a thin‐colored blood flow signal in the true lumen protruding outward (white arrow); the vascular diameter of the intermural hematoma is enlarged. (F) Pulsed wave measures the systolic flow rate of 546 cm/s at the stenosis of the lumen, and the distal blood flow spectrum shows low speed and low pulsation. [Color figure can be viewed at wileyonlinelibrary.com]

**Figure 3:**
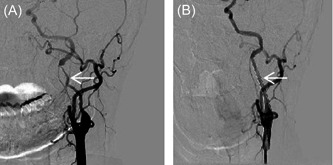
(A) On the left side, digital subtraction angiography shows the dissection from the cervical portion to the petrous portion, where a subtotal occlusion can be seen. The stenosis is about 45 mm long (white arrow). (B) The stent completely covers the stenosis and dissection opening (white arrow).

**Figure 4:**
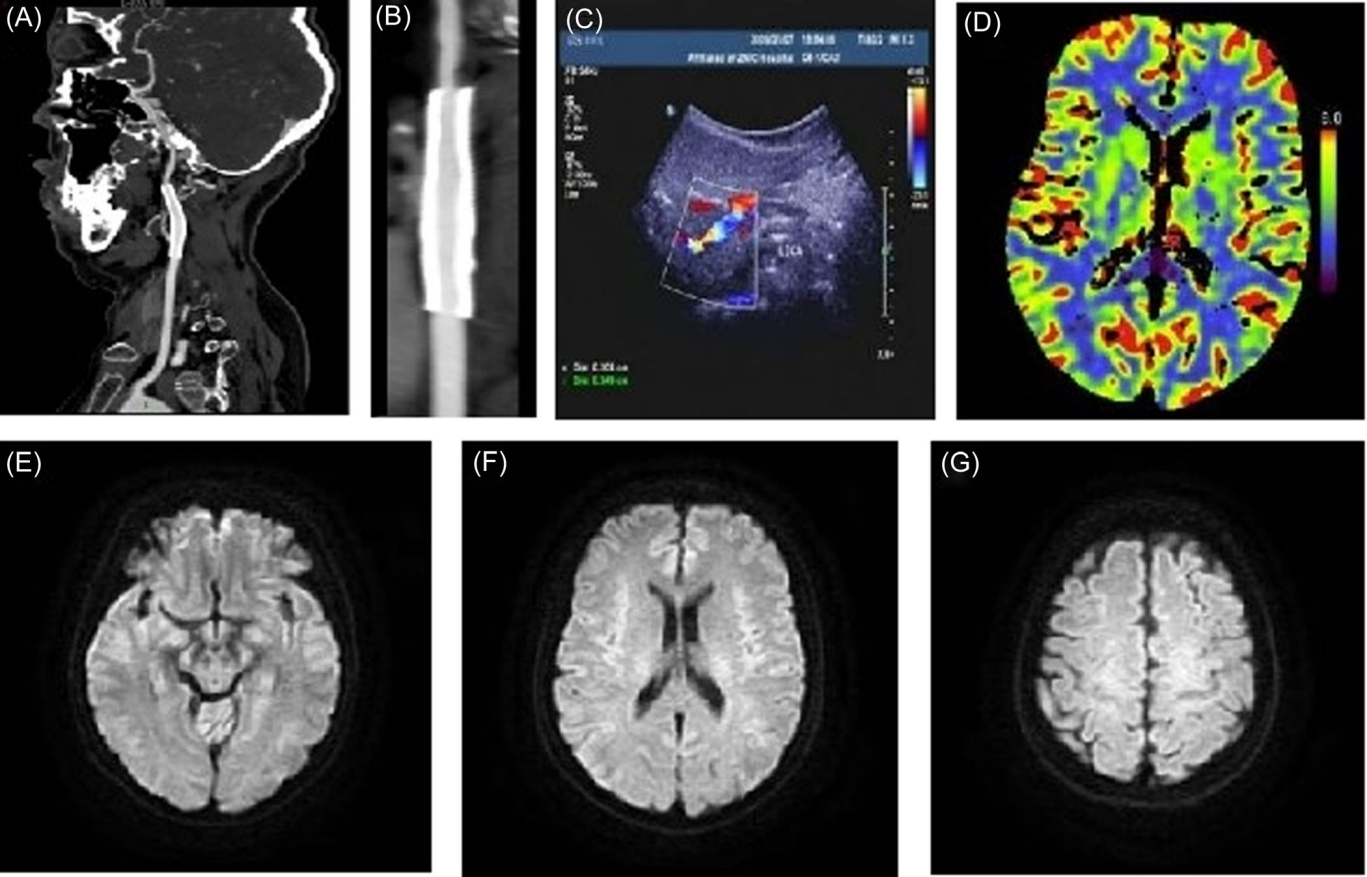
(A, B) Carotid artery computed tomography (CT) angiography. (C) Carotid artery ultrasound showing unobstructed blood flow in the stent. (D) Cranial CT perfusion imaging showing normal cerebral perfusion in both hemispheres. (E–G) Brain magnetic resonance imaging showing no infarction in the left occipital lobe and parietal lobe. [Color figure can be viewed at wileyonlinelibrary.com]

A 56‐year‐old female patient with a 10‐year history of hypertension received long‐term oral metoprolol succinate sustained‐release tablets. Her blood pressure was well controlled, and there were no illegal drug use, smoking, or other specific risk factors for stroke. The patient was sent to the emergency department due to sudden transient aphasia. During a telephone call 10 h ago, she suddenly developed aphasia and stiff tongue movement for about 1 min. Throughout the whole call, she was awake and she felt no discomfort. The blood lipid test results showed that triglyceride was 2.03 mmol/L (normal value <1.7 mmol/L), total cholesterol was 6.82 mmol/L (normal value <5.2 mmol/L), and low‐density lipoprotein cholesterol was 3.71 mmol/L (normal value <3.12 mmol/L). Brain diffusion‐weighted imaging (DWI) results showed multiple acute‐phase small infarcts in the left occipital lobe and parietal lobe (Figure 1A–C), cerebral perfusion imaging showed hypoperfusion of the left cerebral hemisphere (Figure 1D), and no other abnormalities were observed during testing. Combined with clinical symptoms and brain DWI results, the preliminary diagnosis was acute cerebral infarction. The patient received lipid‐lowering treatment with aspirin and atorvastatin. However, after 3 days, the patient developed new symptoms, with repeated hiccups during neck rotation, which disappeared spontaneously after several minutes, and the patient reported intermittent headaches. After a multidisciplinary consultation, further examinations were performed. Cervical magnetic resonance imaging (MRI) showed mild degeneration of the cervical spine, a computerized tomography (CT) scan of the intracranial artery showed that the M3 segment branches of the right middle cerebral artery were reduced, and CT of the carotid artery showed that the segment of the left internal carotid artery was narrowed, so left ICAD was suspected (Figure 2A–C). Carotid ultrasound showed left internal carotid artery dissection (intermural hematoma type), and left internal carotid artery stenosis was 70%–99% (Figures 2D–F). The patient was treated with low‐molecular‐weight heparin anticoagulation and intensive statins. Three days later, the patient developed short‐term weakness of the right limb. We surmised that the formation and detachment of the ICAD mural thrombosis further triggered TIA. To prevent repeated ischemic events and the occurrence of more severe complications, we performed stenting to the stenosis in left ICA with the consent of the patient and her family. Intraoperative digital subtraction angiography (DSA) showed that the left internal carotid artery was dissected from the neck segment to the petrosal segment with a subtotal occlusion, and the stenotic lesion was about 45 mm long (Figure 3A); the stent completely covered the stenosis and dissection opening (Figure 3B). The patient's hiccups disappeared after the operation. To prevent stent thrombosis, the patient was treated with aspirin and clopidogrel for 1 year after discharge, and then switched to aspirin monoclonal antibody treatment. During the 2‐year follow‐up observation, the patient did not show any symptoms such as cerebral ischemia and hiccups. CT angiography (CTA) and carotid artery color Doppler ultrasound showed that the blood flow in the stent was smooth (Figure 4A–C), brain CT scan showed normal perfusion in both cerebral hemispheres (Figure 4D), and brain MRI showed no infarction in the left occipital lobe and parietal lobe (Figure 4E–G).

## 3 DISCUSSION

Typical clinical symptoms of ICAD are unilateral frontotemporal headache, neck pain, and Horner's syndrome.^5^ In this case, the patient showed atypical symptoms; she presented with ischemic stroke, which is more typically seen in young patients with ICAD,^8^ so we ignored the possibility of ICAD when the patient was first admitted. Hypertension and hyperlipidemia were considered as risk factors for ischemic stroke, and cerebral infarction had been treated. After 3 days of treatment, cervical CTA and other examinations of the neck showed that the patient had hiccups when the neck rotated, which led to carotid artery dissection. Poststroke hiccups are common in clinic, usually due to intracranial hemorrhage or infarction, which damages the oblong respiratory center of the medulla oblongata, causing intractable hiccups that are not easy to relieve.^9^, ^10^, ^11^ The medulla oblongata is mainly supplied by the vertebral artery; however, the disease activity score results showed that there were no abnormalities in the vertebral artery, and head MRI results of the head showed that there were no lesions in the medulla oblongata. Therefore, we believed that the hiccups of patients have nothing to do with injury to the medulla oblongata. In addition, some lesions that stimulate the vagus nerve, such as esophagitis, gastric dilation, intestinal obstruction, and infection, may also lead to hiccups.^12^, ^13^ However, our patient had none of the above conditions. Hiccups only occurred when the patient turned her neck, so we speculated that her hiccups might be related to neck lesions. When we placed the stent to restore the normal shape of the blood vessel, the symptoms of hiccups disappeared even when she turned her neck. Therefore, we speculate that the hiccups might be related to the stimulation of the vagus nerve by ICAD.

**Figure 5:**
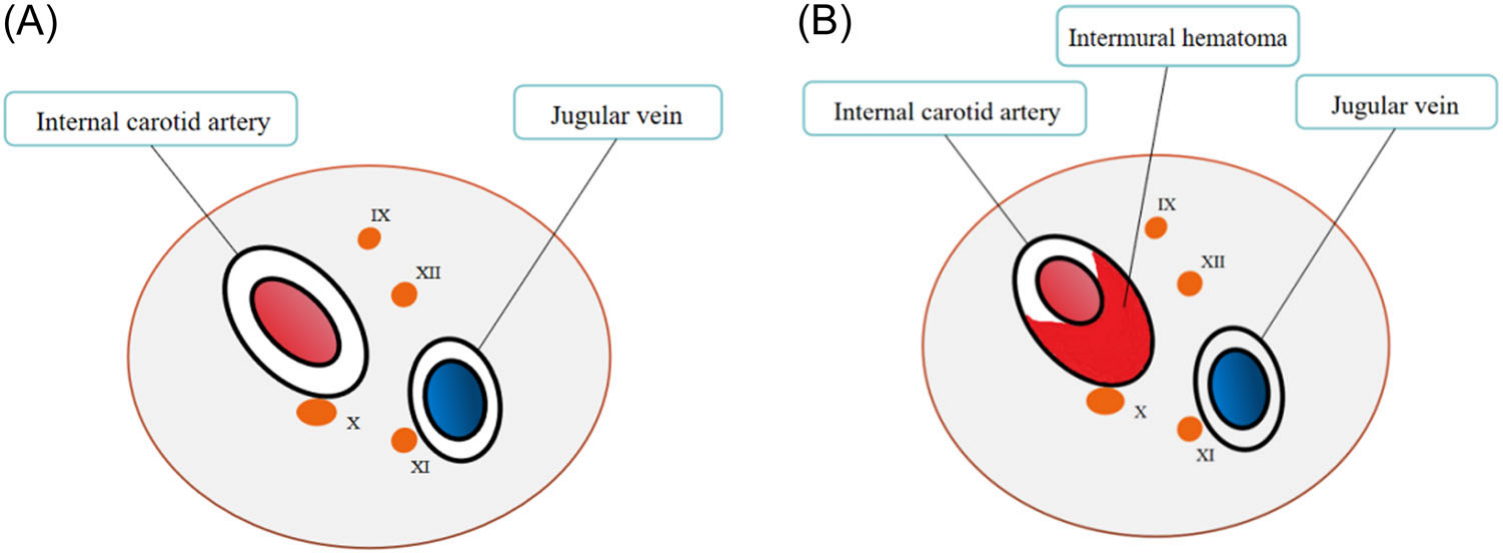
(A) The Xth cranial nerves is in close contact with the internal carotid artery at the level of the atlas. (B) The internal carotid artery is enlarged and stimulates the vagus nerve. [Color figure can be viewed at wileyonlinelibrary.com]

The vagus nerve runs in the carotid sheath and closes contact with the internal carotid artery (Figure 5A). When ICAD occurs, it may affect the adjacent vagus nerve.^14^, ^15^, ^16^, ^17^ Previous case reports have shown that in a few patients with ICAD, cranial nerve injury may occur, which is manifested as a single cranial nerve injury or multiple cranial nerve injuries. Among them, the XII injury of the cranial nerve is the most common, followed by IX, X, and XI. The main mechanism involves the formation of dissecting aneurysm that compresses the posterior cranial nerve, resulting in nerve paralysis.^6^, ^18^, ^19^, ^20^, ^21^, ^22^ In this case, the anatomy of the internal carotid artery resulted in an intermural hematoma, which caused stenosis of the vascular lumen and the shedding of the thrombi, which led to symptoms of ischemic stroke. The patient showed symptoms of posterior cranial nerve injury without the occurrence of aneurysm. It was speculated that although the intermural hematoma of the dissection did not compress the vagus nerve, the hematoma led to dilation of the vascular lumen, and the dilated lumen stimulated the vagus nerve (Figure 5B); especially on rotating the neck, the vagus nerve was further pulled and stimulated, which resulted in repeated hiccups. The patient's hiccups were a manifestation of vagus nerve excitement, which was different from previous reports of posterior cranial nerve palsy. Through medical history examination, we were aware that the patient had a history of repeated severe cough 1 month before admission. Therefore, we speculated that the patient's spontaneous ICAD might be related to severe cough. Cervical hyperextension or excessive rotation of the neck can also cause carotid artery dissection,^23^, ^24^ and it has also been reported that severe hiccups can also cause ICAD,^25^ but the patient did not have hiccup before the onset and hiccups only appeared when the patient turned her neck, so we believe that hiccups may not be the cause of ICAD, but the result of it. When the patient was diagnosed with ICAD, low‐molecular‐weight heparin was used for anticoagulation at first, but the patient developed TIA symptoms after anticoagulation. Considering the poor ineffective anticoagulant therapy, we chose ICAD stent implantation. There was no recurrence of TIA symptoms after the operation. Meanwhile, symptoms such as hiccups and headache disappeared and the patient recovered completely. At present, the treatment of recurrent hiccups caused by ICAD mainly focuses on the cause, that is, the treatment of ICAD. The treatment of ICAD includes thrombolytic therapy, antithrombotic therapy, endovascular therapy, and surgery.^26^, ^27^, ^28^ This case demonstrates that it is difficult to diagnose ICAD without typical clinical manifestations at an early stage, especially in the elderly. In this case, the symptoms of headache and hiccups appeared on the third day of treatment after admission, which indicated that the symptoms of ICAD may evolve and worsen gradually over a few days. Attention should be paid to the changes in the patient's condition at this stage. If there is high suspicion of ICAD during this process, it should be diagnosed by DSA as soon as possible. Although we administered antiplatelet and anticoagulant therapy after admission, the patient still developed TIA, indicating that drug treatment did not prevent further development of the disease. Therefore, we finally chose stent implantation and achieved satisfactory therapeutic effects. We followed up the patients for 2 years after the operation, and the patient did not have hiccups or any symptoms of cerebral ischemia, suggesting that ICAD patients who failed conservative treatment should be treated surgically as soon as possible, and opening narrow or occluded blood vessels can prevent the development of more severe cerebral ischemia.

## 4 CONCLUSION

Because of the lack of atypical symptoms, patients with ICAD often face delay in diagnosis and treatment. In this case, we found that patients with carotid artery dissection may have recurrent hiccups related to neck activity. TIA occurred even after conventional treatment, but the prognosis of early surgical treatment was good. This case suggests that paying attention to the disease progression of high‐risk patients in time will enable us to make an early and effective diagnosis. At the same time, choosing an appropriate treatment plan according to the disease progression of patients will lead to an appropriate prognosis for the patient.

## AUTHOR CONTRIBUTIONS

Zong‐Min Zhang and Man‐Hong Zhou conceptualized the study, drafted this paper, and collected the core data; Li‐Ling provided technical guidance; and Xin‐Xin Yang revised and approved the final version of the article.

## CONFLICT OF INTEREST

The authors declare no conflict of interest.

## ETHICS STATEMENT

This case report was approved by the ethics committee of the Affiliated Hospital of Zunyi Medical University (Approval No: KLL‐2022‐717). Written informed consent was obtained from the patient for publication of this case report.

## Data Availability

The authors confirm that the data supporting the findings of this study are available within the article.
